# Evaluation of Cannabis Use Among US Adults During the COVID-19 Pandemic Within Different Legal Frameworks

**DOI:** 10.1001/jamanetworkopen.2022.40526

**Published:** 2022-11-07

**Authors:** Joshua C. Black, Elise Amioka, Janetta L. Iwanicki, Richard C. Dart, Andrew A. Monte

**Affiliations:** 1 Rocky Mountain Poison & Drug Safety, Denver Health and Hospital Authority, Denver, Colorado; 2 Department of Emergency Medicine, University of Colorado School of Medicine, Aurora

## Abstract

This cross-sectional study analyzes the prevalence of cannabis use by US adults during the COVID-19 pandemic within different legal frameworks and evaluates differences in associated behaviors.

## Introduction

The COVID-19 pandemic caused disruptions to treatment of substance use disorder, the availability of drugs, and other social factors that influence drug use.^1^ In June 2020, more than 10% of adults had started or increased substance use to cope with the pandemic,^2^ which could include using cannabis. The divergence in state-by-state policies governing cannabis sales in the US could create differences in who might use cannabis and for what reasons, particularly because stress can be a catalyst for substance use. In this cross-sectional study, we analyzed changes in prevalence of cannabis use during the pandemic within different legal frameworks and evaluated differences in associated behaviors.

## Methods

In this cross-sectional study, we analyzed the responses to a general population survey^3^ from the Researched Abuse, Diversion and Addiction Related Surveillance System from 2019 to 2021, stratified by prepandemic and pandemic periods. Participants self-reported their sex, age, race and ethnicity, cannabis use in the past 12 months, reason for use, frequency of use in the past 30 days, Drug Abuse Screening Test score, and whether they had used illicit drugs or prescription drugs for nonmedical use (NMU) reasons in the past 12 months. Race and ethnicity data were collected to ensure that aggregate estimates were not influenced by a majority of non-Hispanic White respondents. Cannabis legalization was defined according to availability of recreational or medical sales from dispensaries during survey collection (state-by-state legalization status apepars in the eTable in the Supplement). Weighted prevalence and 95% CIs were estimated using a bootstrap approach.

This study was approved by the Colorado Multiple Institutional Review Board and followed the STROBE reporting guideline. Additional methods appear in the eAppendix in the Supplement.

## Results

A total of 178 824 adults were analyzed; the median age was 51 (IQR, 36-64) years and 53.4% were women. In terms of race and ethnicity, 9.9% of adults were Black, 8.7% were Hispanic, 82.2% were White, and 10.7% reported another race or ethnicity (including American Indian or Alaska Native, Asian, Native Hawaiian or other Pacific Islander, or other race or ethnicity). The prevalence of past 12-month cannabis use in states that had recreational cannabis (hereinafter *recreational states*) was twice that of states in which cannabis was illegal (hereinafter *nonlegal states*) (Table). Prevalence was significantly higher during vs before the pandemic among states that had legalized medical cannabis (hereinafter *medical states*) and nonlegal states; no difference was observed in recreational states. In medical and nonlegal states during the pandemic, higher prevalence was observed among adults aged 18 to 29 and 30 to 54 years vs lower prevalence for adults aged 18 to 29 years in recreational states.

**Table.** Prevalence and Differences in Past 12-Month Use of Cannabis Among US Adults Before and During the COVID-19 Pandemic^a^

Subgroup	Prevalence		Difference^b^
	Before pandemic	During pandemic	
Nonlegal states			
Overall	14.5 (13.3 to 15.7)	18.1 (16.6 to 19.6)	3.6 (1.7 to 5.5)
18-29 y	26.6 (22.7 to 30.4)	33.1 (28.0 to 38.3)	6.6 (0.3 to 12.9)
30-54 y	16.0 (14.2 to 17.8)	21.5 (19.1 to 23.9)	5.5 (2.6 to 8.3)
≥55 y	6.0 (4.8 to 7.1)	6.0 (4.7 to 7.4)	0.1 (−1.6 to 1.7)
Medical states			
Overall	16.3 (15.9 to 16.6)	18.9 (18.6 to 19.3)	2.6 (2.2 to 3.1)
18-29 y	29.2 (28.1 to 30.2)	30.8 (29.6 to 32.0)	1.6 (0.1 to 3.2)
30-54 y	18.1 (17.6 to 18.6)	21.5 (21.0 to 22.1)	3.5 (2.8 to 4.1)
≥55 y	8.8 (8.4 to 9.1)	10.4 (9.9 to 10.8)	1.6 (1.1 to 2.1)
Recreational states			
Overall	27.7 (26.9 to 28.6)	27.8 (27.1 to 28.6)	0.1 (−0.9 to 1.1)
18-29 y	40.1 (38.0 to 42.1)	37.2 (35.3 to 39.2)	−2.8 (−5.5 to −0.1)
30-54 y	28.9 (27.8 to 30.0)	29.9 (28.8 to 31.0)	1.0 (−0.4 to 2.4)
≥55 y	17.1 (16.2 to 18.1)	18.3 (17.5 to 19.1)	1.2 (−0.0 to 2.3)

^a^ Data are presented as percentage (95% CI) of adults.

^b^ Positive difference values indicate that prevalence of cannabis use was higher during the pandemic.

The most common reasons for cannabis use were (1) to relax, reduce stress, or sleep; (2) to get high; and (3) to reduce pain (Figure). During the pandemic, percentages were significantly higher among adults using to relax and reduce pain in medical states and significantly lower among those using to get high and for past 12-month prescription drug NMU. Percentages were significantly lower among those using to get high and for past 12-month prescription drug NMU in recreational states. Changes in nonlegal states were similar to changes in medical states but were nonsignificant.

**Figure.**
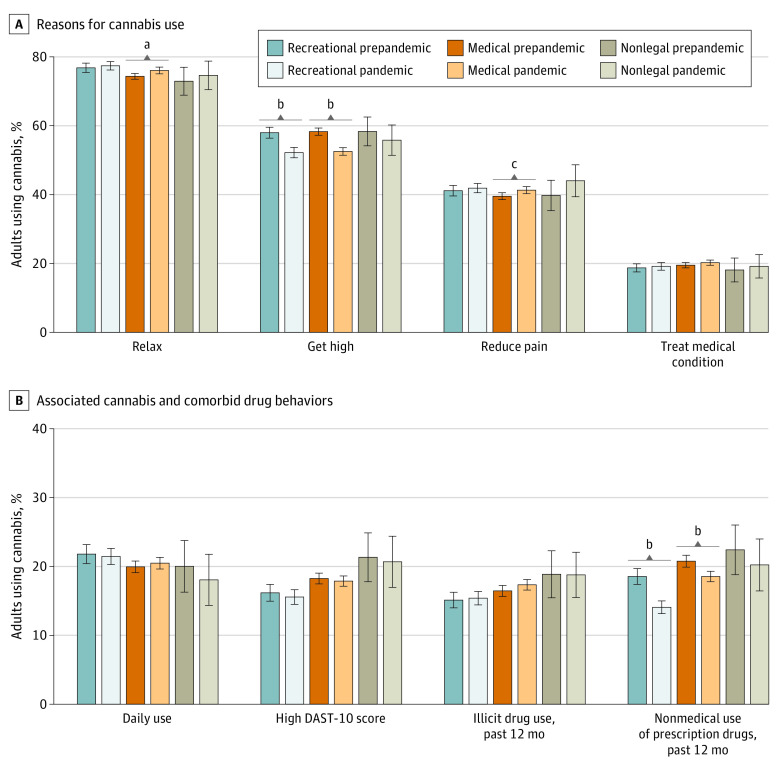
Reasons for Cannabis Use and Associated Behaviors Among US Adults by Legal Status A high Drug Abuse Screening Test (DAST-10) score was defined as 3 or greater. Error bars indicate 95% CIs. ^a^*P* < .01. ^b^*P* < .001. ^c^*P* < .05.

## Discussion

This cross-sectional study found that cannabis use was more prevalent in medical and nonlegal states during vs before the pandemic, but not in recreational states. Other drug use was more frequent during the pandemic.^4^ We observed higher cannabis use to relax and reduce pain, concurrent with decreasing nonmedical use of prescription drugs and use to get high, suggesting that cannabis may have been used to cope with stressors^5^ or compensate for disrupted access to prescription opioids.^6^ The limited variation in percentages suggests that individuals who use cannabis will do so regardless of legality.

A limitation of the study is that the survey did not capture factors preceding cannabis use, individual perceptions of legality, or measures of substance use severity. More restrictive legal frameworks may not prevent substance use during times of stress.

## Notes

Supplement. **eAppendix.** Survey Methodology, Study Variable Definitions, and Statistical Methods **eTable.** Legalization Status of US States During Study Period
eReferences
