# Supplementary material for: Evaluation of Cannabis Use Among US Adults During the COVID-19 Pandemic Within Different Legal Frameworks
Source: JAMA Netw Open. 2022 Nov 7;5(11):e2240526. doi: 10.1001/jamanetworkopen.2022.40526 (PMC9641535; doi:10.1001/jamanetworkopen.2022.40526)
Supplement: Supplement. — eAppendix. Survey Methodology, Study Variable Definitions, and Statistical Methods eTable. Legalization Status of US States During Study Period eReferences [file jamanetwopen-e2240526-s001.pdf]

Supplemental Online Content

Black JC, Amioka E, Iwanicki JL, Dart RC, Monte AA. Evaluation of cannabis use among US adults during the COVID-19 pandemic within different legal frameworks. *JAMA Netw Open*. 2022;5(11):e2240526. doi:10.1001/jamanetworkopen.2022.40526

eAppendix. Survey Methodology, Study Variable Definitions, and Statistical Methods
eTable. Legalization Status of US States During Study Period
eReferences

This supplemental material has been provided by the authors to give readers additional information about their work.

© 2022 Black JC et al. *JAMA Network Open.*

eAppendix: Survey Methodology, Study Variable Definitions, and Statistical Methods

Survey Methodology

The Survey of Non-Medical Use of Prescription Drugs (NMURx) Program from the Researched Abuse, Diversion and Addiction Related Surveillance (RADARS) System employs an online, repeated cross-sectional survey on drug use in the United States. Adult respondents were sampled from a commercial survey panel, and biases were proactively mitigated by implementing best practices during data collection and analysis. Participants consent by agreeing to a consent form before beginning the survey, and they are compensated approximately $2 for completing the survey. Measurement bias was addressed through exclusion criteria to remove respondents providing careless responses. Social desirability bias was addressed by using a confidential online format. Selection bias from the non-probabilistic sampling strategy was addressed by applying calibration weights developed from a combination of demographic and health-related characteristics. All residential 3-digit ZIP codes in the 50 US states and the District of Columbia were represented, mitigating coverage bias. Drug use estimates have good psychometric properties, and results demonstrated high reliability and concurrent validity with three other national surveys.[1] The pre-pandemic data was completely collected in 2019 and in 2020 prior to any public health stay-at-home orders, which first occurred on 19 March 2020 in California (by wave number). The NMURx Program surveys used in this study were collected in 6 waves, with associated pre-pandemic vs during-pandemic definitions, initiated vs eligible responses, and completion rates:

1. 11 March 2019 to 6 June 2019 (pre-pandemic). Initiated: 39,858; eligible: 29,873 (74.9%)
2. 27 September 2019 to 26 November 2019 (pre-pandemic). Initiated: 41,372; eligible: 29,728 (71.9%)
3. 21 January 2020 to 12 March 2020 (pre-pandemic). Initiated: 37,756; eligible: 29,782 (78.9%)
4. 07 July 2020 to 17 September 2020 (pandemic). Initiated: 37,791; eligible: 29,750 (78.7%)
5. 27 January 2021 to 06 March 2021 (pandemic). Initiated: 36,367; eligible: 29,846 (82.1%)
6. 27 August 2021 to 10 October 2021 (pandemic). Initiated: 42,616; eligible: 29,845 (70.0%)

Target sample size for each wave (~30,000) was determined so that national prevalence estimates had a precision of 1-2 percentage points. Panelist may only take the survey once in a calendar year but may return across calendar years.

Study Variable Definitions

Race and ethnicity information were self-identified. Hispanic, White, and Black were reported separately; American Indian, Alaska Native, Asian, Native Hawaiian, Pacific Islander, or another race were summarized together due to infrequent reporting of these categories. Individuals were asked about their reasons for cannabis use and may indicate multiple reasons. The questionnaire included the modified Drug Abuse Screening Test (DAST-10),[2] which evaluates the degree to which participants are experiencing consequences related to drug abuse. High problematic drug use was defined as a DAST-10 score of 3 or higher, which has good sensitivity and specificity for detecting substance use disorder.[3] Comorbid drug use of cannabis with non-medical use (NMU) of prescription drugs was defined as any past 12-month use of cannabis and past 12-month non-medical use of a prescription opioid, stimulant, or sedative; this does not indicate use of both at the same time. NMU of prescription drugs in the past 12 months was defined as "use in a way not directed by your healthcare provider". Comorbid drug use of cannabis with use of illicit drugs was defined as any past 12-month use of cannabis and past 12-month use of drugs not made by a drug company and not obtained from a doctor, including: amphetamine, cocaine, crack, fentanyl, GHB, heroin, LSD, MDMA, mescaline, methamphetamine, PCP, or psilocybin.

Recreational cannabis states were defined as having both recreational and medical cannabis legalized with dispensaries during the study period. Medical only states were defined as states that had active medical cannabis dispensaries, and non-legal states had neither medical nor recreational dispensaries available during the study period (see eTable for designations). Six states (Arizona, Arkansas, Illinois, Louisiana, Maine, Michigan) changed their dispensing frameworks during the study period, and responses from these states were defined by whether medical or recreational dispensaries (or both) were open during the time the waves were collected. The eTable denotes which waves were stratified into each legal framework.

© 2022 Black JC et al. *JAMA Network Open.*

Statistical Methods

Weights were calculated using generalized raking,[3] and all estimates were calculated using these weights. To account for the non-probability sampling and correlation from respondents taking the survey in multiple calendar years, standard errors and 95% confidence intervals were calculated from 250 bootstrap replicates, where individuals were resampled with replacement. The p-values for differences in proportions were calculated from z-scores derived from the estimates and bootstrapped standard errors. Analyses were conducted using SAS 9.4 (Cary, NC).

© 2022 Black JC et al. *JAMA Network Open.*

eTable: Legalization Status of US States During Study Period

Non-Legal Cannabis States	Medical-Only Cannabis States	Recreational Cannabis States
Arkansas (Wave 1)<br>Idaho<br>Indiana<br>Kansas<br>Louisiana (Wave 1)<br>Nebraska<br>South Dakota	Alabama<br>Arizona (Waves 1-4)<br>Arkansas (Waves 2-6)<br>Connecticut<br>Delaware<br>Florida<br>Georgia<br>Hawaii<br>Illinois (Waves 1-2)<br>Iowa<br>Kentucky<br>Louisiana (Waves 2-6)<br>Maine (Waves 1-5)<br>Maryland<br>Michigan (Waves 1-4)<br>Minnesota<br>Mississippi<br>Missouri<br>Montana<br>New Hampshire<br>New Jersey<br>New Mexico<br>New York<br>North Carolina<br>North Dakota<br>Ohio<br>Oklahoma<br>Pennsylvania<br>Rhode Island<br>South Carolina<br>Tennessee<br>Texas<br>Utah<br>Vermont<br>Virginia<br>West Virginia<br>Wisconsin<br>Wyoming	Alaska<br>Arizona (Waves 5-6)<br>California<br>Colorado<br>Illinois (Waves 3-6)<br>Maine (Wave 6)<br>Massachusetts<br>Michigan (Waves 5-6)<br>Nevada<br>Oregon<br>Washington<br>District of Columbia

eReferences

1. Black JC, Rockhill K, Forber A, et al. An Online Survey for Pharmacoepidemiological Investigation (Survey of Non-Medical Use of Prescription Drugs Program): Validation Study. J Med Internet Res. Oct 25 2019;21(10):e15830. doi:10.2196/15830
2. McCabe SE, Boyd CJ, Cranford JA, Morales M, Slayden J. A modified version of the Drug Abuse Screening Test among undergraduate students. J Subst Abuse Treat. 2006;31(3):297-303. doi:10.1016/j.jsat.2006.04.010
3. Yudko E, Lozhkina O, Fouts A. A comprehensive review of the psychometric properties of the Drug Abuse Screening Test. J Subst Abuse Treat. Mar 2007;32(2):189-98. doi:10.1016/j.jsat.2006.08.002

© 2022 Black JC et al. *JAMA Network Open.*
